# Advancements in vaccine development and therapeutic approaches for severe fever with Thrombocytopenia Syndrome

**DOI:** 10.3389/fcimb.2025.1650652

**Published:** 2025-09-23

**Authors:** Tsenddari Ganbold, Satabdi Acharya, Sunirmal Sheet, Enkhtsatsral Munkhtur, Seong-Tshool Hong

**Affiliations:** ^1^ Department of Biomedical Sciences and Institute for Medical Science, Jeonbuk National University Medical School, Jeonju, Republic of Korea; ^2^ Animal Genomics and Bioinformatics Division, Rural Development Administration, National Institute of Animal Science, Wanju, Republic of Korea

**Keywords:** severe fever with thrombocytopenia syndrome (SFTS), vaccine candidates, animal models of SFTSV, therapeutic approaches, antiviral strategies

## Abstract

Severe Fever with Thrombocytopenia Syndrome (SFTS), caused by the SFTS virus (SFTSV), is a tick-borne disease that poses a significant public health threat due to its high fatality rate and the lack of effective treatments or vaccines. Since its first identification, SFTSV has been reported in multiple countries, including China, South Korea, Japan, and Vietnam, highlighting its expanding regional footprint and potential global impact. This review summarizes recent preclinical and early clinical advancements in vaccine development and therapeutic strategies for SFTSV, with an emphasis on novel vaccine platforms and antiviral approaches. It also discusses the critical role of immunocompromised animal models, such as IFNAR^–^/^–^ and STAT2^–^/^–^ mice, in elucidating disease mechanisms and evaluating candidate interventions. Promising vaccine platforms include mRNA-based vaccines, viral vector vaccines, protein subunit vaccines, and inactivated virus vaccines. Additionally, therapeutic approaches—such as RNA-dependent RNA polymerase (RdRp) inhibitors, calcium channel blockers, immunotherapies, targeted therapeutic strategies, and traditional Chinese medicine—have demonstrated potential in preclinical and limited clinical studies. However, challenges remain, including reduced efficacy when treatment is administered late, limited validation in human clinical trials, and the risk of viral resistance. Despite progress, key challenges persist in modeling human SFTS in animals and translating preclinical findings to clinical applications. This review underscores the urgent need for continued research to address these gaps, accelerate the development of effective countermeasures, and enhance global preparedness for future SFTSV outbreaks.

## 1 Introduction

**Figure 1:**
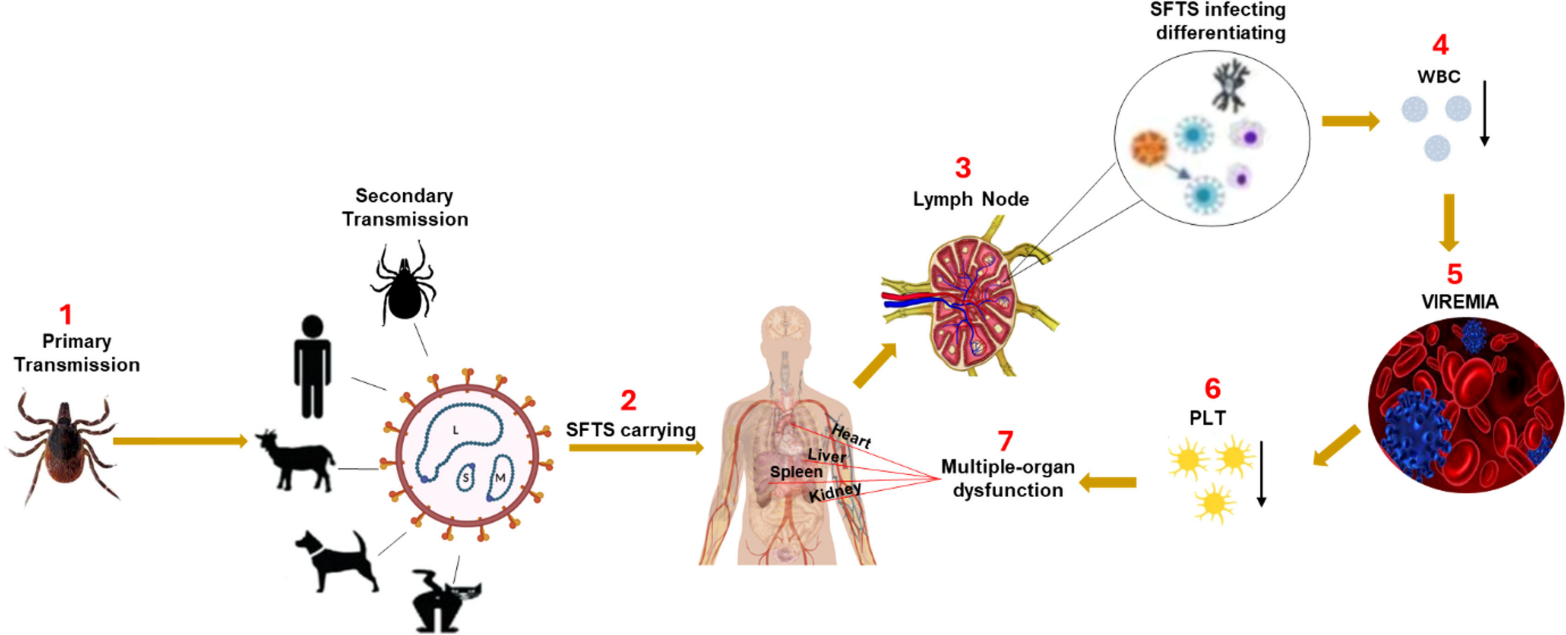
The mechanism of SFTSV pathogenesis. SFTSV is primarily transmitted to humans through the bite of an infected *Haemaphysalis longicornis* tick. After entry, the virus initially infects immune cells in the local lymph nodes near the bite site, including mononuclear phagocytic cells (macrophages and dendritic cells) and B-cells. Early viral replication in these immune cells impairs the host immune response, causing a reduction in white blood cells (WBC), particularly leukopenia and lymphocytopenia, hallmark features of SFTSV infection. As the virus spreads via the bloodstream (viremia), it triggers excessive immune activation, resulting in a cytokine storm characterized by the rapid release of key cytokines such as TNF-α, IL-6, and IFN-γ. This exaggerated inflammatory response leads to severe inflammatory response syndrome (SIRS) and widespread tissue damage. The cytokine storm plays a central role in multi-organ failure by inducing endothelial dysfunction, increasing vascular permeability, and promoting disseminated intravascular coagulation, which together cause organ ischemia and damage (Liu et al., 2014). The vascular endothelium is particularly susceptible to cytokine-induced injury, compromising blood vessel integrity. Platelets (PLT) adhere to damaged vessel walls and aggregate, contributing to thrombocytopenia. The cytokine storm and ensuing immune dysregulation cause multi-organ dysfunction, especially affecting the liver, kidneys, and lungs, potentially leading to fatal complications if not managed promptly.

Severe Fever with Thrombocytopenia Syndrome (SFTS) is a tick-borne viral infection caused by the SFTSV, a member of the Phenuiviridae family. First identified in China in 2009, the disease has since spread to South Korea, Japan, and other East Asian countries. SFTSV, a new tick-borne viral disease with a high mortality rate in Asia, was first reported in China in 2009, South Korea in 2010, Japan in 2013, Vietnam in 2017, Myanmar in 2018, Taiwan in 2019, and Thailand and Pakistan in 2020 (Kida et al., 2019; Yamanaka et al., 2020). SFTSV is primarily transmitted through bites from infected *Haemaphysalis longicornis* ticks (**Figure 1**), although human-to-human transmission has also been documented, particularly in healthcare settings through direct contact with bodily fluids of infected individuals (Reece et al., 2018; Zhuang et al., 2018; Casel et al., 2021). In addition, animal-to-human transmission has been reported, especially through close contact with infected domestic animals such as cats and dogs bite (Tsuru et al., 2021), veterinarians infected through contact with a sick cat (Miyauchi et al., 2022) and epidemiological cases in Japan involving saliva exposure from companion animals in the absence of tick bites (Kobayashi et al., 2020). Due to its high fatality rate, ranging from 30% to 50%, and reported cases in over 10 countries, including China, South Korea, and Japan, SFTSV continues to pose a significant public health threat, further exacerbated by the lack of effective treatments (Park et al., 2019; Casel et al., 2021; Cho et al., 2021).

The clinical manifestations of SFTSV range from mild flu-like symptoms to severe complications, including multiorgan failure and hemorrhagic symptoms (Lambert and Gernsheimer, 2017). Despite extensive research, there are currently no approved antiviral treatments or vaccines for SFTSV. Ongoing studies have explored various therapeutic strategies, developed animal models to study disease progression, and investigated promising vaccine candidates (Yu et al., 2024). Reliable animal models are crucial for understanding SFTSV pathogenesis and developing effective interventions. Immunocompromised mouse models, such as type I interferon receptor knockout (IFNAR^–^/^–^) mice, have been vital for studying SFTSV infection due to the limited susceptibility of wild-type mice (Sun et al., 2022; Kim et al., 2024). Non-human primate (NHP) models, particularly macaques, offer a closer representation of human disease due to their similar immune system, making them invaluable in preclinical evaluations (Grow et al., 2016; Lemaitre et al., 2021). Other models, including ferrets and hamsters, are also being explored for their potential to advance SFTSV research (**Table 1**) (Park et al., 2019).

**Table 1:** Overview of the current SFTS animal model and potential vaccines.

Animal model		Animal survival	Vaccine candidates	Reference
*Mouse*	Adult C57BL/6,BALB/c,Kunming,ICR(CD-1),A/J, CAST/EiJ,DBA/1J,FVB/NJ, NZBWF1/J,BXD68/RwwJ, BXD34/TyJ,SJL/J,C3H, FVB,129S1/svlmJ	Non-lethal	Whole inactivated vaccine Protein subunit vaccine	(Chen et al., 2012; Jin et al., 2012; Kaneko et al., 2018; Park et al., 2020; Li et al., 2022)
	Newborn C57BL/6,BALB/c,Kunming,ICR (CD-1)	Lethal		(Chen et al., 2012; Ning et al., 2019)
	Mitomycin C-treated C57BL/6	Lethal		(Jin et al., 2012)
	IFNAR Ab-treated C57BL/6	Lethal		(Park et al., 2020)
	IFNAR^-^/^-^ C57BL/6,129/Sv	Lethal	DNA vaccine Viral vectored vaccine Protein subunit vaccine mRNA vaccine	(Liu et al., 2014; Tani et al., 2016; Matsuno et al., 2017; Westover et al., 2019; Yoshikawa et al., 2019; Kang et al., 2020)
	Aged C57BL/6,BALB/c,129S1/svlmJ	Non-lethal		(Matsuno et al., 2017; Park et al., 2019)
	STAT1^-^/^-^ C57BL/6	Non-lethal		(Yoshikawa et al., 2019)
	STAT2^-^/^-^ C57BL/6	Lethal		(Yoshikawa et al., 2019)
	Pregnancy C57BL/6	Non-lethal		(Chen et al., 2020)
	Ips-1^-^/^-^ C57BL/6	Non-lethal		(Yamada et al., 2018)
	Myd88^-^/^-^ C57BL/6	Lethal		(Yamada et al., 2018)
	Ips-1^-^/^-^Myd88^-^/^-^ C57BL/6	Non-lethal		(Yamada et al., 2018; Mo et al., 2020)
	MOV10 KD C57BL/6	Non-lethal		(Yamada et al., 2018)
	Humanized Human CD34 C HSC-transferred mouse, human PBMCs-transferred mouse (HuPBL–NCG mouse)	Lethal		(Li et al., 2019; Wu et al., 2020)
*Rat*	Adult Wistar rat	Non-lethal		(Chen et al., 2012)
	Newborn Wistar rat	Lethal		(Chen et al., 2012)
*Non-human primate*	Adult Rhesus macaque, Cynomolgus macaque	Non-lethal		(Jin et al., 2015; Matsuno et al., 2017)
*Hamster*	Adult Syrian golden hamster	Non-lethal		(Chen et al., 2012; Jin et al., 2012; Liu et al., 2014)
	Newborn Syrian golden hamster	Non-lethal		(Chen et al., 2012; Liu et al., 2014)
	STAT2^-^/^-^ Syrian golden hamster	Lethal		(Gowen et al., 2017)
*Ferret*	Adult (≤2 years old) Mustela putorius furo	Non-lethal		(Gowen et al., 2017; Yun et al., 2020)
	Aged (≥4 years old) Mustela putorius furo	Lethal	Live-attenuated vaccine DNA vaccine Protein subunit vaccine	(Yu et al., 2019; Kwak et al., 2020; Yun et al., 2020)
*Cat*	Adult (2 years old) Newborn (0.5 year old) Russian Blue, American Shorthair	Lethal (4 of 6 cats)		(Park et al., 2019)

Efforts to develop vaccines against SFTSV have gained momentum in recent years, with promising advancements across several platforms. In preclinical studies, mRNA-based vaccines, modeled after successful COVID-19 vaccines, have shown strong immunogenicity and protective efficacy (Hashimoto et al., 2022; Lelis et al., 2023). Viral vector vaccines utilizing adenovirus and vesicular stomatitis virus (VSV) platforms have demonstrated robust immune responses (Wu et al., 2014). Additionally, protein subunit vaccines using recombinant viral glycoproteins have elicited protective neutralizing antibody responses. In clinical stages, inactivated virus vaccines are being investigated for their potential effectiveness (**Table 2**) (Zhou et al., 2006; Liu et al., 2023; Zhang et al., 2024).

**Table 2:** Summary of vaccine platform features.

Vaccine platform	Immunogenicity	Scalability	Storage requirements	Reference
*mRNA Vaccines*	Elicits strong humoral (neutralizing antibodies) and cellular (CD4^+^ and CD8^+^ T cell) immune responses. Induces rapid antibody production post-prime. Easily modifiable for emerging viral variants through sequence updates. Demonstrated efficacy against diverse pathogens including SARS-CoV-2. Minimal risk of genomic integration.	Highly scalable due to *in vitro*, cell-free production processes. Does not require biological materials like eggs or mammalian cells. Enables rapid manufacturing, easy standardization, and high batch-to-batch consistency. Suitable for pandemic response due to fast turnaround from design to deployment.	Requires cold-chain logistics. Traditional LNP-mRNA vaccines need ultra-cold storage (-80 °C to -20 °C). Newer formulations (e.g., thermostable LNPs or lyophilized forms) show stability at 2–8 °C for short durations. Ongoing development toward room-temperature-stable mRNA vaccines.	(Sahin et al., 2014; Pardi et al., 2018b; Zhang et al., 2019)
*Viral Vector Vaccines*	Induce strong and durable humoral (neutralizing antibodies) and cellular (CD8^+^ cytotoxic and CD4^+^ helper T cell) immune responses. Capable of inducing immunity after a single dose. Use of replication-deficient vectors (e.g., adenovirus) enhances safety. However, efficacy may be reduced due to pre-existing immunity to the viral vector, especially common serotypes (e.g., Ad5). New strategies like using rare or chimpanzee-derived vectors are being explored to overcome this.	Moderately scalable. Requires cell-based production systems (e.g., HEK293 or PER.C6 cells) and complex bioprocessing. Longer production timelines and higher biosafety requirements than mRNA. Scale-up feasible but slower and more costly compared to *in vitro* platforms.	Most formulations are stable at 2–8 °C, facilitating distribution in standard cold-chain conditions. Some candidates require freezing (≤ -20 °C) for extended storage. Efforts are ongoing to improve thermostability, including lyophilized formulations.	(Pardi et al., 2018b; Logunov et al., 2020; Zhu et al., 2020)
*Protein Subunit Vaccines*	Elicit strong humoral (antibody) responses, particularly against the targeted protein antigen. Often require adjuvants (e.g., alum, AS01, MF59) to enhance immunogenicity and induce cellular responses. CD4^+^ T cell activation is typically observed, but CD8^+^ responses are limited. Established safety profile due to non-replicating and non-infectious components. Multiple doses usually required for optimal protection.	Moderate to high scalability. Based on recombinant protein production in systems such as yeast, insect, or mammalian cells (e.g., CHO, Sf9). Production infrastructure is well-established globally. However, manufacturing is slower and more complex than synthetic platforms like mRNA. Downstream purification and quality control can be time-consuming.	Generally stable at 2–8 °C, making them well-suited for global distribution with standard refrigeration. Some formulations exhibit long shelf-life, and thermostable or lyophilized versions are under development to enhance deployment in resource-limited settings	(Lee and Nguyen, 2015; Heath et al., 2021; Liang et al., 2021)

Although no specific antiviral therapy for SFTSV has been approved, multiple treatment strategies have been explored. Favipiravir and ribavirin have shown antiviral activity *in vitro* and in limited clinical settings, although their effectiveness remains uncertain. In addition, therapeutic approaches targeting the RNA-Dependent RNA Polymerase (RdRp) of SFTSV, calcium channel inhibitors that may disrupt viral entry or replication, immunotherapy strategies aimed at modulating host immune responses, targeted therapy approaches based on understanding viral-host interactions, and traditional Chinese medicines with potential antiviral properties are being actively investigated. Supportive care, including fluid replacement, blood transfusions, and organ support, remains the primary approach to managing SFTSV complications.

Despite these efforts, significant gaps remain between promising preclinical findings and their successful clinical application, underscoring the need for further research to bridge these gaps and translating these approaches into effective treatments.

## 2 The structure and genome of the SFTSV

SFTSV is an enveloped virus, meaning that it possesses a lipid membrane derived from the host cell. This envelope plays a crucial role in viral entry by enabling fusion with host cell membranes. Embedded in the envelope are two glycoproteins, Gn and Gc, which form spike-like structures on the viral surface and mediate attachment to and entry into host cells (Yu et al., 2011). The structural components, particularly the glycoproteins and the nonstructural NSs protein, are essential for viral infectivity and immune evasion. Gn and Gc interact with host cell surface receptors, initiating endocytosis and subsequent fusion of the viral envelope with the endosomal membrane. After entry, the viral ribonucleoprotein complexes (RNPs) comprising the viral RNA segments encapsidated by the nucleoprotein (N) are released into the cytoplasm for replication and transcription. The NSs protein is a key virulence factor involved in immune evasion. It antagonizes host interferon signaling by targeting and inhibiting STAT proteins and TBK1, both of which are central to the interferon-mediated antiviral response. By suppressing these pathways, SFTSV can replicate efficiently while evading immune detection (Jiao et al., 2013; Du et al., 2023). SFTSV belongs to the *Bandavirus* genus of the *Phenuiviridae* family within the order *Bunyavirales*. Its genome consists of three single-stranded RNA segments: two negative-sense and one ambisense. These segments designated L, M, and S encode different viral proteins. The L segment encodes the RNA-dependent RNA polymerase (L protein), the M segment encodes the glycoproteins Gn and Gc, and the S segment encodes both the nucleoprotein (N) and the nonstructural protein (NSs). The L protein comprises multiple functional domains, including a cap-binding domain, an endonuclease domain, and an RNA-dependent RNA polymerase (RdRp) domain. These domains mediate critical processes such as host mRNA cap-snatching, viral transcription, and replication during the viral life cycle (Yu et al., 2011; Vogel et al., 2020). The L segment of the SFTSV genome encodes RNA-dependent RNA polymerase (RdRp), a key enzyme in viral replication and transcription. RdRp comprises three functional domains: an endonuclease, a polymerase core, and a cap-binding domain. Through a mechanism called cap snatching, the cap-binding domain binds host mRNA, and the endonuclease cleaves it to generate a short, capped RNA fragment. This fragment serves as a primer for the polymerase core to initiate viral RNA synthesis. This strategy enables efficient replication and mRNA production while conserving the virus’s genetic resources (Chen et al., 2020; Vogel et al., 2020; Wang et al., 2020; Malet et al., 2023). The M segment of SFTSV encodes two envelope glycoproteins, Gn and Gc, which are essential for viral entry into host cells. These proteins mediate attachment to host cell receptors particularly DC-SIGN on dendritic cells and macrophages triggering endocytosis and membrane fusion. Gn and Gc undergo post-translational modifications, such as glycosylation, which enhance their stability, receptor-binding ability, and immune evasion. They are also key targets for neutralizing antibodies, which can block infection. By exploiting immune cells for entry and dissemination, SFTSV establishes systemic infection while evading early immune detection (Lozach et al., 2011; Hofmann et al., 2013). The S segment of the SFTSV genome encodes the nucleoprotein (N) and nonstructural protein (NSs). The N protein encapsidates viral RNA, protecting it during replication and transport, while NSs suppresses the host interferon (IFN) response, aiding immune evasion and viral replication. N also interacts with RdRp to form the replication and transcription complex (RNP). The mRNAs for N and NSs are transcribed from sense and antisense RNA strands, respectively, allowing regulated protein expression. After viral entry via receptors like DC-SIGN, the genome is released, transcribed by RdRp, and translated into viral proteins, supporting efficient replication and infection (Eifan and Elliott, 2009; Walter et al., 2011; Lokupathirage et al., 2021). The newly synthesized N protein binds viral RNA to form protective N–RNA complexes, essential for genome packaging and transport to assembly sites. In the cytoplasm, viral components including RNA, glycoproteins, and structural proteins assemble into complete virions. These virions bud from the plasma membrane, acquiring a lipid envelope with embedded Gn and Gc proteins, forming infectious particles. This efficient use of host cellular machinery enables SFTSV replication, assembly, and continued spread within the host (Liu et al., 2020; Williams et al., 2023).

## 3 Animal models of SFTSV

### 3.1 Immunocompetent adult mice

C57BL/6 mice were infected with SFTSV (HB29 strain, 10^5^ TCID_50_) via intramuscular injection. Shortly after infection, the mice exhibited distinct abnormalities associated with SFTSV, including leukocytopenia, thrombocytopenia, and elevated levels of ALT, AST, and BUN. Remarkably, all mice survived the infection, and no clinical symptoms such as fever or body weight loss were observed. These findings highlight the limitations of the C57BL/6 model in accurately recapitulating severe human disease, particularly the fatal progression of SFTSV.

The absence of severe symptoms in this model underscores the need for improved animal systems that better replicate the pathophysiology of SFTSV in humans an essential step for developing effective treatments and vaccines with translational relevance. Viral replication was detected only in the spleen, suggesting that this organ may be the primary target in this model. However, it remains unclear whether this observation reflects human disease pathogenesis or is a species-specific feature of mice.

In humans, SFTSV infection is associated with multi-organ involvement, including the liver and kidneys, which may not be fully represented in this mouse model. Transient pathological lesions observed during the 28-day infection period resolved without intervention. In the early stages of infection, the spleen and bone marrow exhibited the most notable pathological changes. Specifically, there was an increase in megakaryocytes (the primary hematopoietic cells) in both organs, while lymphocyte populations in the red pulp of the spleen were reduced (Jin et al., 2012). Collectively, the adult C57BL/6 model reflects some aspects of human SFTSV infection but presents a major limitation in that it does not progress to severe illness or mortality. Similarly, other immunocompetent adult mouse strains, including BALB/c, C3H, FVB, and ICR (CD-1), also fail to exhibit severe clinical symptoms or lethality following SFTSV infection (Jin et al., 2012; Matsuno et al., 2017; Park et al., 2019).

### 3.2 Immunocompromised adult mice

Immunocompromised adult mice such as those lacking the interferon-α/β receptor (IFNAR^–^/^–^), STAT2 knockout mice (STAT 2^–^/^–^), or mice treated with IFNAR-blocking antibodies (IFNAR Ab) or mitomycin C have been widely used to study SFTSV infection. These models effectively bypass the innate immune restrictions that typically limit SFTSV pathogenicity in wild-type mice. As a result, they enable a more accurate replication of human SFTSV disease progression, which is otherwise challenging due to the natural resistance of immunocompetent mice.

As expected, these immunocompromised animals exhibit pronounced clinical signs of severe illness following SFTSV challenge, including weight loss, thrombocytopenia, leukocytopenia, elevated viral loads in serum and tissues, and increased susceptibility to viral replication and dissemination. Moreover, these models have proven instrumental in evaluating the *in vivo* efficacy of candidate antiviral drugs, therapeutic antibodies, and vaccine platforms.

The consistent development of severe disease in these models makes them valuable tools for mechanistic studies of SFTSV pathogenesis as well as for preclinical testing of therapeutic strategies (Chen et al., 2012; Jin et al., 2012; Liu et al., 2014; Matsuno et al., 2017; Westover et al., 2019; Yoshikawa et al., 2019; Park et al., 2020; Perez-Sautu et al., 2020; Kim et al., 2023).

#### 3.2.1 Mitomycin C-treated mice (increased disease severity)

Mitomycin C suppresses the immune system by inhibiting DNA synthesis in proliferating cells, thereby impairing the host’s ability to mount an effective immune response. By targeting rapidly dividing immune cells, mitomycin C reduces both innate and adaptive immune functions, creating an environment more permissive to viral replication and dissemination. Consequently, this weakened immune state results in more severe disease outcomes, as infected hosts are less capable of controlling viral replication, regulating inflammation, and preventing systemic spread of the virus.

In a representative study, C57BL/6 mice received daily intraperitoneal injections of mitomycin C (0.02 mg per mouse) from three days before to three days after exposure to SFTSV (10^5^ TCID_50_, intramuscular). Compared to untreated controls, these mice exhibited markedly more severe clinical symptoms, including significant weight loss, reduced mobility, and ruffled fur—hallmarks of systemic illness. Within 10 days post-infection, 50% of the mitomycin C-treated mice succumbed to the infection, underscoring the critical role of intact host immunity in surviving SFTSV. Surviving mice experienced substantial and prolonged weight loss, indicating a lasting disease burden even in the absence of lethality.

These findings further support the value of immunocompromised models, such as mitomycin C-treated mice, for studying SFTSV pathogenesis and evaluating candidate therapeutics under conditions of suppressed host immunity (Jin et al., 2012).

#### 3.2.2 IFNAR^–^/^–^ mice (a widely used model for acute fatal disease)

IFNAR^–^/^–^ mice, which lack functional type I interferon (IFN-α/β) receptors, are highly susceptible to SFTSV infection and exhibit severe clinical symptoms, including significant weight loss, thrombocytopenia, leukocytopenia, and high viremia. These features make them a widely used and valuable animal model for studying the pathogenesis of acute and often fatal SFTSV disease. Due to their heightened sensitivity, this model is also extensively employed to evaluate the safety, immunogenicity, and protective efficacy of candidate vaccines, antiviral drugs, and immunotherapies in preclinical studies.

However, several key limitations of the IFNAR^–^/^–^ mouse model must be carefully considered when interpreting experimental results. Most notably, these mice entirely lack the initial antiviral defense typically mediated by type I interferons a critical component of the innate immune response. As a result, IFNAR^–^/^–^ mice may not fully replicate the complex virus–host interactions seen in immunocompetent individuals, where early IFN signaling plays a central role in controlling infection. Moreover, these mice often develop severe symptoms rapidly, with high mortality occurring within a short time frame post-infection. This rapid disease course limits their utility for studying the later stages of SFTSV, such as viral persistence, chronic immune dysregulation, or delayed tissue pathology, which are relevant in some human cases.

Therefore, while the IFNAR^–^/^–^ mouse model is particularly effective for rapidly assessing therapeutic interventions under highly susceptible conditions, it is not ideal for exploring the full spectrum of SFTSV pathogenesis or for modeling the more gradual and heterogeneous clinical outcomes observed in humans. Complementary models such as those with partial immune suppression or humanized immune systems may be necessary to achieve a more comprehensive understanding of SFTSV pathophysiology (Tani et al., 2016; Matsuno et al., 2017; Westover et al., 2019; Park et al., 2020).

#### 3.2.3 STAT2^–^/^–^ and STAT1^–^/^–^ mice (examining the role of STAT signaling in SFTSV infection)

STAT1^–^/^–^ and STAT ^–^/^–^mouse models have been employed to evaluate host susceptibility to SFTSV infection and to elucidate the distinct roles of specific interferon signaling components in viral control and pathogenesis. In a comparative study, the infectivity and pathogenicity of SFTSV were assessed in STAT1^–^/^–^, STAT ^–^/^–^, IFNAR^–^/^–^, and wild-type (WT) mice. Following viral challenge, both STAT2^–^/^–^ and IFNAR^–^/^–^, mice exhibited significant weight loss, leukocytopenia (reduced white blood cells), and thrombocytopenia (reduced platelets) between 4- and 8-days post-infection. The severity and progression of clinical symptoms were comparable between the STAT2^–^/^–^ and IFNAR^–^/^–^, groups, suggesting that disruption of STAT2-mediated signaling has effects like complete ablation of type I IFN receptor signaling.

On the other hand, STAT1^–^/^–^ mice experienced a milder disease course, showing only transient symptoms, and all animals survived the infection. Wild-type mice, used as controls, remained entirely asymptomatic. The survival of STAT1^–^/^–^ mice, despite the absence of STAT1, suggests the presence of compensatory antiviral mechanisms possibly through redundant interferon signaling pathways or alternative antiviral defenses that partially control viral replication.

Further virological analysis showed that viral titers in organs such as the spleen and liver of STAT 2^–^/^–^ mice were comparable to those in IFNAR^–^/^–^, mice and significantly higher than those in STAT1^–^/^–^ mice. This finding highlights the particularly critical role of STAT2 and its downstream antiviral genes in restricting SFTSV replication.

In summary, these results demonstrate that although both STAT1 and STAT2 contribute to interferon signaling, STAT2 plays a more non-redundant and essential role in controlling SFTSV infection. Consequently, STAT 2^–^/^–^ mice serve as a valuable model for studying severe disease phenotypes and for evaluating therapeutic strategies targeting viral replication and immune modulation (Yoshikawa et al., 2019).

### 3.3 Newborn and aged mice (age-related susceptibility in mice)

Previous experimental results indicate that newborn mice from various genetic backgrounds, including KM, BALB/c, and C57BL/6 strains, are highly susceptible to Hantaan virus (HYSV) infection. When challenged via intracardiac injection with a dose of 2×10^7^ viral copies per animal, all newborn mice succumbed to the infection, resulting in a uniformly fatal outcome for this route and dosage. However, when exposed to a slightly higher viral load (3×10^7^ copies per animal) via intraperitoneal injection, the mortality rate decreased notably, ranging from 35% to 50%, suggesting that the route of viral administration plays a significant role in determining disease severity.

Similarly, newborn Wistar rats displayed high susceptibility to HYSV infection. Following intracardiac inoculation with 2×10^7^ copies per animal, 100% mortality was observed. In comparison, when challenged intraperitoneally with a higher dose of 5×10^7^ copies per animal, the mortality rate was reduced to approximately 40%. This pattern, mirroring that observed in mice, underscores the importance of the infection route in modulating disease severity in neonatal rodents.

Interestingly, newborn hamsters exhibited a starkly different response. Neither intracardiac nor intraperitoneal administration of high viral loads (2×10^7^ copies intracardiac and 5×10^7^ copies intraperitoneal per animal) led to any fatalities. These findings suggest species-specific resistance to HYSV infection in neonatal hamsters, possibly due to inherent biological differences such as immune system maturity or receptor availability, which may provide protection against fatal outcomes.

Collectively, these results emphasize that susceptibility to HYSV infection varies significantly across rodent species, as well as between species and developmental age. Moreover, the route of infection profoundly influences mortality rates, with intracardiac inoculation generally leading to higher lethality compared to intraperitoneal challenge. Understanding these species- and age-dependent differences is essential for selecting appropriate animal models for studying HYSV pathogenesis, immune responses, and evaluating potential antiviral therapies (Chen et al., 2012).

### 3.4 Hamsters (resistance and lethal models with STAT2 deficiency)

The Golden Syrian hamster, the most used laboratory hamster strain, exhibits notable resistance to Severe Fever with Thrombocytopenia Syndrome Virus (SFTSV) infection when exposed via various routes, including intravenous, intramuscular, intraperitoneal, and intracerebral administration (Jin et al., 2012; Liu et al., 2014). This resistance is observed in both adult and newborn hamsters, as newborns inoculated with SFTSV do not experience more severe illnesses compared to their adult counterparts. This finding contrasts with other animal models, where neonatal susceptibility is typically heightened. It is hypothesized that hamsters, regardless of age, may possess inherent immunological or cellular characteristics that limit the severity of SFTSV infection. Potential mechanisms could include a robust and rapid innate immune response, variations in pattern recognition receptors (PRRs), differences in antiviral signaling pathways, or alterations in cellular receptors critical for SFTSV entry and replication. For instance, hamster cells may lack, or express lower levels of specific surface molecules required for efficient viral binding and entry, thereby restricting infection at an early stage.

Further studies are needed to elucidate the specific molecular and immunological factors underlying this species-specific resistance. Understanding these mechanisms could provide valuable insights into host-pathogen interactions and facilitate the development of novel therapeutic strategies or the identification of potential antiviral targets.

Although adult hamsters largely resist overt clinical illness, histopathological examinations have revealed mild, transient changes in organs such as the liver and kidneys following SFTSV infection. These changes include slight inflammation, hepatocellular degeneration, and minor renal tissue alterations, typically resolving them without leading to clinical symptoms. This transient organ pathology suggests that while the virus induces localized cellular stress or immune activation, the hamster immune system effectively controls and clears the infection without progression to severe disease.

To create a more susceptible hamster model for studying SFTSV pathogenesis, STAT2-deficient (STAT2^–^/^–^) hamsters were developed. These animals lack a key transcription factor in the type I and type III interferon signaling pathways, rendering them highly vulnerable to viral infections. When infected with SFTSV, STAT2^–^/^–^ hamsters displayed symptoms like those observed in human cases, including fever, significant weight loss, and notable organ damage, particularly in the liver and spleen. However, a notable difference was the absence of significant leukocytopenia, a common clinical hallmark in human SFTSV cases. Interestingly, in the context of another emerging viral pathogen, a novel Roborovski hamster strain (SH101) has been reported to closely mimic systemic SARS-CoV-2 infection in humans, exhibiting hallmark features such as respiratory distress, fever, weight loss, and multi-organ involvement. This demonstrates how alternative hamster strains can serve as valuable preclinical models for studying viral pathogenesis and evaluating vaccines or antivirals (Zhai et al., 2021).

Using the STAT2^–^/^–^ model, therapeutic interventions were evaluated. Favipiravir, an RNA polymerase inhibitor, demonstrated complete protection against lethal SFTSV infection when administered to these hamsters, highlighting its strong antiviral efficacy. In contrast, ribavirin, another nucleoside analog with broad-spectrum antiviral activity, failed to protect STAT2^–^/^–^hamsters, suggesting limited effectiveness against SFTSV in the absence of interferon signaling (**Table 1**) (Park et al., 2020). These findings underscore the importance of host innate immune responses in controlling SFTSV infection and provide a valuable preclinical model for evaluating vaccines and antiviral agents.

### 3.5 Rats (limited use and future directions for modeling SFTSV infection)

Compared to other laboratory rodents, rats are not commonly used as a standard model for studying SFTSV infection. However, available evidence suggests that newborn rats can develop fatal Severe Fever with Thrombocytopenia Syndrome in a manner like that observed in newborn mice. In experimental infections, newborn Wistar rats (1–3 days old) exposed to SFTSV through the intracardiac route at a dose of 2 × 10^7^ copies per animal uniformly succumbed to the infection within 7 days post-inoculation, indicating high susceptibility and lethality at an early developmental stage. Similarly, when newborn rats were infected via the intraperitoneal route with a slightly higher dose (3 × 10^7^ copies per animal), approximately 40% of the animals died, suggesting that both the route of infection and viral dose significantly influence disease severity and mortality rates.

In stark contrast, all adult Wistar rats infected with SFTSV, whether via intracardiac injection (3 × 10^7^ copies) or intraperitoneal injection (1.25 × 10^8^ copies), survived the infection and exhibited no obvious clinical symptoms during a 25-day post-infection observation period. An effective results indicate that adult rats, like adult mice and hamsters, possess a more mature and effective immune response capable of controlling SFTSV replication and preventing severe disease outcomes.

Despite these observations, the characterization of SFTSV infection in rats remains incomplete. To date, detailed histopathological and hematological examinations in both newborn and adult rats following SFTSV infection have not been thoroughly documented. Such studies are crucial for understanding organ-specific pathology, immune responses, and potential subclinical effects of the virus in this species. Furthermore, information regarding viral loads in various tissues, cytokine responses, and the kinetics of viral clearance in rats is still lacking. These gaps highlight the need for more comprehensive investigations to determine the utility of rat models, especially for studying age-dependent differences in SFTSV pathogenesis and for evaluating antiviral treatments or vaccine candidates in the future (**Table 1**) (Chen et al., 2012).

### 3.6 Ferrets (semi-susceptibility and age-dependent disease severity)

Young adult (≤ 2 years of age) and aged (≥ 4 years of age) ferrets, scientifically known as Mustela putorius furo, were administered an intravenous injection containing 10^7^ TCID_50_/mL of the SFTSV SPL010 strain in a fixed volume of 0.5 mL. Following inoculation, the ferrets were closely monitored over a two-week observation period to assess clinical symptoms, hematological changes, and viral replication dynamics. The young adult ferrets were found to be semi-susceptible to SFTSV infection, displaying only mild clinical manifestations, including transient weight loss and low-grade fever. Hematological analysis showed that key parameters, such as platelet (PLT) count, white blood cell (WBC) count, aspartate aminotransferase (AST), and alanine aminotransferase (ALT) levels, remained within normal ranges throughout the infection period. Despite the mild symptoms, viral RNA was detectable in several organs, suggesting that while systemic viral dissemination occurred, the young adult ferrets mounted an effective immune response that limited disease severity. All young adult ferrets recovered without fatalities after 16 days of monitoring, demonstrating their relative resilience to SFTSV infection.

On the other hand, aged ferrets exhibited significantly more severe disease outcomes. Clinical observations revealed a higher mortality rate among aged animals, alongside pronounced clinical symptoms such as sustained fever, progressive weight loss, thrombocytopenia (reduced platelet count), leukopenia (reduced white blood cell count), and elevated serum ALT and AST levels markers indicative of liver damage. Hematological assessments confirmed a marked decline in WBC and PLT counts, correlating with impaired immune function and increased vulnerability to infection. Furthermore, viral RNA was extensively detected in multiple tissues, including spleen, liver, lungs, and kidneys, providing evidence of widespread systemic infection. Histopathological examinations, although not detailed in the study, would likely reveal tissue inflammation and organ dysfunction, further contributing to the fatal outcomes observed in the aged ferret group.

The underlying hypothesis for the observed age-dependent differences in disease severity suggests that the higher mortality rate in older ferrets may result from a combination of factors, including age-associated immune senescence and the substantial viral inoculum used. Immunosenescence, characterized by diminished innate and adaptive immune responses, likely hampers the ability of aged ferrets to control viral replication effectively, leading to unchecked systemic infection and organ failure. Notably, no deaths occurred among the young adult ferrets, emphasizing the protective effect of a more robust immune system in younger animals.

These findings underscore the importance of considering host age when modeling SFTSV infection and evaluating potential therapeutic interventions. The ferret model particularly aged ferrets provides a valuable platform for studying severe disease phenotypes and assessing the efficacy of antiviral drugs and vaccine candidates targeting vulnerable populations (Park et al., 2019).

### 3.7 Non-human primates

Thus far, two reports have examined the impact of SFTSV infection on non-human primate models, specifically rhesus macaques (Macaca mulatta) (Matsuno et al., 2017) and cynomolgus macaques (Macaca fascicularis) (Malet et al., 2023). Cynomolgus macaques infected with the SFTSV SD4 strain at a dose of 10^6^ TCID_50_ via the subcutaneous route exhibited no visible clinical signs throughout the monitoring period. Viremia was undetectable in their blood samples, and histopathological examination of major organs revealed no significant abnormalities or tissue damage. These findings suggest that cynomolgus macaques are either inherently less susceptible to SFTSV infection or that the infection in this species remains subclinical, characterized by effective immune control that prevents symptomatic disease. Consequently, cynomolgus macaques may not be an ideal model for studying severe SFTSV pathogenesis but could be useful for studying asymptomatic or mild infections.

In comparison, rhesus macaques inoculated with the SFTSV HB29 strain at a higher dose of 10^7^ TCID_50_ via the intramuscular route developed clear signs of illness. Clinical observations included the onset of fever, a significant reduction in platelet counts (thrombocytopenia), and decreased white blood cell counts (leukocytopenia) hallmarks commonly associated with human SFTSV cases. Despite these clinical symptoms, all infected rhesus macaques recovered spontaneously without the need for antiviral treatment, suggesting that while rhesus macaques are more susceptible to SFTSV infection than cynomolgus macaques, the infection typically follows a mild to moderate course.

Histopathological analysis further highlighted the effects of SFTSV infection in rhesus macaques. Liver tissues exhibited notable abnormalities, including multifocal mild-to-moderate piecemeal necrosis, bridging necrosis of hepatocytes, and mild hepatocellular degeneration. Inflammatory cell infiltration was also observed within liver tissues, indicating an active immune response to viral replication. These hepatic changes are consistent with clinical reports of liver involvement in human SFTSV cases, where hepatic dysfunction is a common complication.

The differential susceptibility and pathological outcomes between cynomolgus and rhesus macaques underscore the importance of selecting appropriate non-human primate models for SFTSV research. Rhesus macaques, showing moderate clinical disease and histopathological changes, represent a more suitable model for studying SFTSV pathogenesis, immune responses, and therapeutic interventions than cynomolgus macaques. However, the relatively mild disease severity observed even in rhesus macaques suggests that further refinement of the infection model-such as using higher viral doses, different viral strains, or immunosuppressive pre-treatment-might be necessary to fully replicate the severe disease spectrum observed in human SFTSV patients.

Future studies are warranted to better understand the mechanisms underlying species-specific differences in susceptibility, as well as to optimize non-human primate models for preclinical evaluation of SFTSV vaccines and antiviral therapies.

### 3.8 Cats

Four out of six cats infected with SFTSV (SPL010 strain, 10^7^ TCID_50_ via intravenous route) developed clinical signs of illness, demonstrating a high degree of susceptibility to the virus. The infected cats exhibited symptoms that closely mirrored those seen in human patients with severe cases of SFTSV, including fever, leukocytopenia (a decrease in white blood cells), thrombocytopenia (low platelet count), marked weight loss, anorexia, jaundice, and general depression. These symptoms were consistent with the acute, systemic nature of the disease, highlighting the cats’ vulnerability to SFTSV infection.

Beyond the common clinical manifestations, all infected cats showed significant hemophagocytosis a pathological process in which immune cells engulf and digest other blood cells particularly within their spleen, bone marrow, lymph nodes, and liver. This process, which plays a critical role in the development of SFTSV in humans, was more pronounced in the cats, suggesting an exaggerated immune response. Furthermore, the cats exhibited severe damage to their gastrointestinal tract, including hemorrhage in the mucosa, as well as necrosis (tissue death) and inflammatory cell infiltration. This gastrointestinal damage closely resembled the pathological changes seen in humans but was notably more severe in the feline models.

Pathological examinations of the cats that succumbed to the infection revealed significant hemorrhaging within the gastrointestinal mucosa, as well as severe necrotic lesions and hemorrhage in lymphatic tissues such as the spleen and lymph nodes. Inflammation was widespread in these tissues, suggesting that the infection had triggered a robust but damaging immune response. These pathological findings are consistent with those observed in fatal human cases of SFTSV, providing further evidence that cats develop a disease progression similar to that of human patients.

The prominent hemophagocytosis observed in various organs, particularly in the spleen, bone marrow, lymph nodes, and liver underscores its crucial role in disease pathogenesis. This phenomenon, often associated with severe viral infections, reflects an overactive immune response that contributes to tissue damage and organ dysfunction. In addition to the gastrointestinal damage and lymphatic infiltration, the widespread immune activation and organ damage observed in these cats make them an ideal model for studying the systemic effects of SFTSV and the immunopathology of SFTSV.

Overall, cats represent a highly promising animal model for SFTSV research due to their high susceptibility to infection and the remarkable similarities in clinical, hematological, and histopathological manifestations to severe human SFTSV cases. Their response to SFTSV infection closely mirrors the disease progression in humans, making them a valuable tool for studying the pathophysiology of the disease, as well as for testing potential therapeutic interventions. The insights gained from studies using cats could significantly improve our understanding of SFTSV infection and contribute to the development of more effective vaccines and treatments for this fatal disease (Park et al., 2019).

## 4 SFTSV vaccine candidates

### 4.1 Live-attenuated vaccine

A live-attenuated vaccine is a type of vaccine created by reducing the virulence of a pathogen while maintaining its viability (live). These vaccines are designed to elicit a strong and long-lasting immune response without causing the actual disease (Matsuno et al., 2017; Park et al., 2019; Malet et al., 2023). Live-attenuated vaccines mimic natural infections more closely than other vaccine types, stimulating both humoral and cellular immunity. They are often effective with fewer doses and can provide long-term protection. However, they must be carefully designed to ensure safety, especially in immunocompromised individuals, as even attenuated pathogens could pose risks to such hosts.

In the context of Severe Fever with Thrombocytopenia Syndrome, a study investigated the potential of using live-attenuated SFTSV strains as vaccine candidates (Westover et al., 2019). Two recombinant viruses, rHB2912aaNSs and rHB29NSsP102A, were engineered using a reverse genetics system from the HB29 strain of SFTSV. These recombinant strains involve targeted mutations in the NSs (nonstructural) protein, a key virulence factor known to antagonize the host’s innate immune response. Specifically, rHB2912aaNSs contains an internal deletion of the NSs protein (amino acids 2–282), preserving only the N-terminal methionine and the C-terminal 11 amino acids. Meanwhile, rHB29NSsP102A includes a point mutation, substituting proline at position 102 with alanine. These genetic alterations reduce the virus’s ability to suppress interferon responses and potentially attenuate its pathogenicity.

The NSs protein of SFTSV plays a central role in immune evasion by inhibiting the TPL2 signaling pathway and the secretion of interferon-β (IFN-β), both of which are essential for initiating antiviral defense mechanisms. The two recombinant viruses with disrupted NSs function failed to suppress these immune pathways, leading to reduced replication and disease-causing ability in experimental models. These findings strongly support the potential of NSs-mutated viruses as live-attenuated vaccine candidates, capable of inducing protective immunity while minimizing safety concerns (Brennan et al., 2017; Choi et al., 2019; Choi et al., 2020). Animal studies have demonstrated that these recombinant SFTSVs elicit immune responses characterized by the production of neutralizing antibodies and activation of T-cell responses, without inducing severe symptoms in immunocompetent mice. Importantly, vaccinated animals challenged with wild-type SFTSV exhibited significant protection, further validating the efficacy of these live-attenuated vaccine constructs.

Despite these promising results, translating live-attenuated SFTSV vaccines into clinical use will require extensive preclinical safety evaluations. These include assessing viral stability, reversion risks, and host range limitations. Moreover, studies in larger, more physiologically relevant animal models, such as STAT2^–^/^–^ hamsters or non-human primates, are necessary to better understand the protective mechanisms and potential risks.

Live-attenuated vaccines represent a promising avenue for SFTSV prevention due to their strong immunogenicity and potential for long-lasting protection. However, their development must balance efficacy with stringent safety considerations, especially for use in vulnerable populations.

### 4.2 DNA vaccine

A DNA vaccine is a type of vaccine that uses a piece of genetically engineered DNA to stimulate an immune response. This approach involves introducing a specific gene encoding an antigen (a protein from the pathogen) directly into the body’s cells. The decision to prioritize a DNA vaccine for SFTSV, rather than more traditional platforms or recent mRNA vaccine advancements, may stem from several factors, including the ease of large-scale production, stability at ambient temperatures, and the ability to generate strong cellular immune responses. Additionally, DNA vaccines are less susceptible to issues like mRNA instability and require less complex manufacturing infrastructure, which is crucial in resource-limited settings or for rapid deployment during outbreaks (Vartak and Sucheck, 2016; Kozak and Hu, 2023).

The M segment, responsible for encoding Gn and Gc, has been selected as a promising DNA vaccine candidate for further research and development. This decision was made after recognizing the presence of neutralization epitopes primarily located in the viral glycoprotein Gn, which plays a crucial role in viral attachment and entry into host cells, making it a prime target for inducing neutralizing antibodies. Additionally, Gc, N, NSs, and RdRp were included to generate a broader immune response. Gc, as the second envelope glycoprotein, complements Gn by aiding in viral fusion, while N and NSs are involved in viral replication and immune evasion. RdRp, the viral polymerase, is critical for viral genome replication. Together, these components were selected to provide a comprehensive immune defense against SFTSV.

DNA plasmids containing the complete Gn, Gc, N, NSs, and RdRp genes of SFTSV were synthesized and named pVax1-Gn, pVax1-Gc, pVax1-N, pVax1-NSs, and pVax1-RdRp. These plasmids utilize the human cytomegalovirus (CMV) immediate-early enhancer and promoter to drive gene expression. To assess the effectiveness of the vaccine, mature ferrets (aged over 4 years) were immunized with a combination of all five SFTSV DNA vaccines (pVax1-Gn, pVax1-Gc, pVax1-N, pVax1-NSs, and pVax1-RdRp) via intradermal injection. This vaccination process was repeated three times at two-week intervals, followed by *in vivo* electroporation at the injection site. The ferrets were fully protected from a challenge with 10^7.6^ TCID_50_ of SFTSV, administered intramuscularly two weeks after the last vaccination. In contrast, the control group, which received the control plasmid backbone (modified pVax1), exhibited a peak viral load of 10^4^ copies/mL serum on the fourth day after infection. The viral load of SFTSV in the serum of ferrets that received the SFTSV DNA vaccines remained undetectable for eight days after the challenge.

Additional research indicated that a combination of envelope glycoproteins (pVax1-Gn and pVax1-Gc) or non-enveloped proteins (pVax1-N, pVax1-NSs, and pVax1-RdRp) provided complete protection against fatal SFTSV infection in ferrets. In naïve ferrets, the transfer of sera from those immunized with pVax1-Gn and pVax1-Gc successfully protected against fatal infection, unlike sera from mice immunized with pVax1-N, pVax1-NSs, and pVax1-RdRp. Notably, immunization with only pVax1-N, pVax1-NSs, or pVax1-RdRp significantly decreased vaccine effectiveness, suggesting that a combination of at least two or three of these non-enveloped proteins is essential for full protection (Kwak et al., 2020).

In an alternative research project focusing on DNA vaccine development, vectors were constructed to encode Gn, Gc, N, and NSs, with the addition of mIL-12 to boost cellular immunity. mIL-12 is a cytokine known to enhance the immune response by stimulating T cell activation and promoting the differentiation of naïve T cells into Th1 cells. This Th1 response is crucial for effective cellular immunity, as it enhances the activity of cytotoxic T lymphocytes (CTLs) and natural killer (NK) cells, both of which are essential for eliminating virus-infected cells. Similar strategies involving cytokines like IL-12 have been employed in other viral vaccine development projects to improve the magnitude and quality of the immune response, leading to more robust protection against pathogens. For instance, the use of IL-12 in DNA vaccines for influenza and HIV has shown promising results in enhancing cellular immunity and improving vaccine efficacy.

IFNAR KO mice were administered the DNA vaccine through electroporation at weeks 0, 2, and 4, with a dosage of 4 μg. It was observed that the DNA vaccine, when lacking mIL-12, was unable to stimulate the production of SFTSV N-recognizing IgG. This highlights the significance of mIL-12 in effectively activating the immune response. Following a lethal infection with 1 × 10^5^ focus-forming units of SFTSV, mice immunized with the Gn, Gc, N, and NSs DNA vaccine along with mIL-12 exhibited complete protection against fatality. Additionally, they showed partial protection against body weight loss, thrombocytopenia, and serum viremia. In contrast, mice that received the Gn, Gc, N, and NSs DNA vaccine without mIL-12 exhibited only a 40% survival rate, along with significant body weight reduction, thrombocytopenia, and detectable levels of virus in the bloodstream, indicating inadequate immunogenicity for conferring protection against fatal infection.

These findings suggest that further refinement is necessary for DNA vaccine candidates to confer adequate immune protection in vaccinated animals. However, the inclusion of mIL-12 raises safety concerns, as it could potentially lead to adverse immune reactions, particularly in the context of autoimmune or inflammatory conditions. To address these concerns, ongoing strategies include optimizing mIL-12 dosage, adjusting the timing of administration, and exploring alternative immune adjuvants or delivery systems that may mitigate adverse effects while enhancing immune responses. Additionally, studies are underway to evaluate the safety profiles of cytokine-based adjuvants and their long-term impact on immune tolerance and inflammation in animal models (**Table 1**) (Kang et al., 2020).

### 4.3 Whole inactivated viral vaccine

Whole inactivated viral vaccines are a type of vaccine made from viruses that have been killed or inactivated so they cannot replicate or cause disease. Despite being inactivated, these viruses still elicit an immune response, helping to protect against future infections with the live virus (Vartak and Sucheck, 2016; Choi et al., 2020). This strategy has been widely used in vaccine development for various viral diseases due to its favorable safety profile and proven ability to induce protective immunity.

In this study, a complete β-propiolactone (BPL)-inactivated viral vaccine was developed. BALB/c and C57BL/6 mice were immunized intramuscularly with different doses (0.25, 1, or 4 μg) of inactivated SFTSV, based on the Gn/Gc glycoprotein levels. Some mice received the vaccine with aluminum hydroxide (Al(OH)_3_) adjuvant, while others did not. Immunizations were administered at weeks 0, 2, and 4. Among the mice, those immunized with the highest dose (4 μg) of inactivated SFTSV with Al(OH)_3_ showed the strongest IgG response against the SFTSV N protein and the highest serum neutralization titer against the HB29 strain of SFTSV. However, anti-N IgG responses and serum neutralization titers were similar for the other doses, regardless of the presence of the adjuvant.

C57BL/6 mice that had been immunized were subsequently infected with 10^5^ TCID_50_ of SFTSV two weeks after their final immunization. Mice that received the highest dose of inactivated SFTSV with adjuvant displayed the most notable enhancement in viral clearance. However, no statistically significant difference was observed among the various doses and adjuvant groups, suggesting a potential plateau in the immune response or limited sensitivity in detecting subtle differences in this model.

Nevertheless, due to the constraints of using wild-type (WT) BALB/c and C57BL/6 mouse models, which do not exhibit overt clinical symptoms when infected with SFTSV, the range of immunogenicity of the whole inactivated SFTSV vaccine is limited to stimulating humoral and cellular immunity (Liu et al., 2020). These limitations hinder a complete assessment of the vaccine’s protective efficacy in preventing clinical disease manifestations, such as fever, thrombocytopenia, or organ damage, which are characteristic of human SFTSV cases.

To overcome these challenges, more susceptible animal models, such as aged ferrets, immunocompromised mice, or non-human primates, may provide a more robust evaluation of protective efficacy, especially in mimicking severe clinical symptoms and immune-pathological outcomes. In addition, exploring heterologous prime-boost strategies, optimizing the formulation, or incorporating more immunogenic antigens such as glycoprotein subunits (Gn or Gc) may enhance the protective capacity of inactivated vaccines. In summary, while the inactivated SFTSV vaccine demonstrates immunogenic potential, further studies are required to refine its formulation and validate its effectiveness in disease-relevant models before progressing to clinical development.

### 4.4 Viral vector vaccine

A viral vector vaccine uses a modified virus (the vector) to deliver genetic material from a pathogen (such as a virus or bacterium) into the body’s cells. This genetic material instructs the cells to produce a protein (antigen) from the pathogen, which then triggers an immune response (Matsuno et al., 2017). In this study, a complete β-propiolactone (BPL)-inactivated viral vaccine was developed. BALB/c and C57BL/6 mice were immunized intramuscularly with different doses (0.25, 1, or 4 μg) of inactivated SFTSV, based on the Gn/Gc glycoprotein levels. Some mice received the vaccine with aluminum hydroxide (Al(OH)_3_) adjuvant, while others did not. Immunizations were administered at weeks 0, 2, and 4. Among the mice, those immunized with the highest dose (4 μg) of inactivated SFTSV with Al(OH)_3_ showed the strongest IgG response against the SFTSV N protein and the highest serum neutralization titer against the HB29 strain of SFTSV. However, the anti-N IgG responses and serum neutralization titers were similar for the other doses, regardless of the presence of the adjuvant.

C57BL/6 mice that had been immunized were subsequently infected with 10^5^ TCID_50_ of SFTSV two weeks after their final immunization. Mice that received the highest dose of inactivated SFTSV with adjuvant showed the most notable enhancement in viral clearance. However, no statistically significant differences were observed among the various doses and adjuvant groups, suggesting that while humoral immune responses were induced, protective efficacy may plateau beyond a certain dose or adjuvant threshold.

Due to the limitations of using wild-type (WT) BALB/c and C57BL/6 mouse models, which do not exhibit symptoms when infected with SFTSV, the immunogenicity of the whole inactivated SFTSV vaccine was limited to stimulating humoral and cellular immunity. This limitation hinders a complete evaluation of the vaccine’s efficacy in mimicking the full spectrum of clinical disease. Therefore, while these findings demonstrate that the vaccine is immunogenic and can promote partial viral control, they do not allow a thorough assessment of the vaccine’s ability to prevent clinical disease manifestations such as fever, thrombocytopenia, or organ damage.

Alternative animal models, such as immunocompromised mice (e.g., IFNAR^–^/^–^ or STAT2^–^/^–^), hamsters, or non-human primates, may be more suitable for recapitulating the severe symptoms of SFTSV infection. These models not only display clinical signs but also allow for more detailed evaluations of histopathological changes and systemic viral dissemination. In particular, STAT2^–^/^–^ hamsters have been shown to mimic human SFTSV pathology and could offer a more robust platform for assessing the protective efficacy and safety of vaccine candidates, including whole inactivated virus formulations.

Furthermore, future research may focus on optimizing the antigen formulation, exploring different adjuvant combinations (such as toll-like receptor agonists or saponin-based adjuvants), and adjusting immunization schedules to enhance both the breadth and durability of the immune response. Importantly, long-term protection and the ability to neutralize multiple SFTSV strains or variants remain key goals for advancing this vaccine platform toward clinical application.

### 4.5 rVSV-based vaccines

rVSV-based vaccines use a recombinant vesicular stomatitis virus (rVSV) as a vector to deliver genetic material encoding antigens from a target pathogen. The vesicular stomatitis virus (VSV) is an animal virus that has been engineered to express proteins from other viruses, thereby eliciting an immune response against those proteins (Rodriguez et al., 2019; Robbins et al., 2022; Ahata and Akçapınar, 2023).

In this investigation, a recombinant vesicular stomatitis virus (rVSV) expressing Gn/Gc from the AH12 strain of SFTSV (rVSV-SFTSV/AH12-GP) was developed. C57BL/6 and IFNAR KO mice were immunized intraperitoneally with 2 × 10^4^ focus-forming units (FFU) of different rVSV vectors carrying glycoproteins from EBOV, Hantaan virus (HTNV), or SFTSV to assess the virulence of the vaccine candidates. The results indicated that rVSV vectors carrying VSV or EBOV glycoproteins caused 100% fatality by three- and five-days post-immunization, respectively, exhibiting virulence in IFNAR KO mice. In contrast, rVSV-SFTSV/AH12-GP, where the inclusion of SFTSV/AH12-GP reduced rVSV growth in culture, did not cause body weight loss or fatality in IFNAR KO mice. Immunized IFNAR KO mice were then exposed to 21 × 10^4^ FFU of SFTSV to assess protective immunity against serum viremia, body weight loss, and fatality. Mice that received either rVSV-SFTSV/AH12-GP or rVSV expressing HRTV Gn/Gc (rVSV-eGFP-HRTV-GP) were completely shielded from symptoms upon challenge with SFTSV or HRTV, indicating cross-protection. These results not only demonstrate successful defense against lethal SFTSV infection with a single dose but also highlight the broad cross-protection provided by rVSV-SFTSV/AH12-GP and rVSV-eGFP-HRTV-GP vaccines against both HRTV and SFTSV infections. Given these promising results in mice, the potential for advancing this rVSV-based platform into human clinical trials or primate studies is significant. If further validation in larger animal models is successful, this platform could offer a promising approach for SFTSV and other related viral infections, warranting consideration for future human clinical studies (Dong et al., 2019).

#### 4.5.1 rVAC-based vaccines

rVAC-based vaccines use a recombinant vaccinia virus (rVAC) as a vector to deliver genes encoding antigens from target pathogens. The vaccinia virus, a member of the poxvirus family, has historically been used in the smallpox vaccine. It is well-suited for recombinant vaccine development due to its large genome, which allows for the insertion of foreign genes, and its ability to elicit strong immune responses (Belyakov et al., 2003; Morikawa et al., 2005; Koch et al., 2020).

In this study, the researchers utilized a recombinant m8 vaccinia virus to express different forms of the SFTSV virus. These forms included the N glycoproteins, with and without N, which were then assembled into virus-like particles (VLPs). The study involved subcutaneously immunizing IFNAR KO mice with varying doses of the m8-N, m8-GPC, or m8-N+GPC vaccines at specific time intervals. The results showed that all vaccine candidates effectively induced the production of IgG antibodies that recognized the SFTSV N protein or glycoproteins. However, only the m8-GPC and m8-N+GPC vaccines were able to generate SFTSV-neutralizing antibodies, with no significant difference between them. Furthermore, when the IFNAR KO mice were exposed to lethal infections of the YG-1 strain of SFTSV, those immunized with any of the three vaccines were fully protected from weight loss and fatality. In a separate *in vivo* experiment, IFNAR KO mice were first vaccinated with the Lister strain VACV, a vaccine used for defense against smallpox, and then vaccinated with m8-N, m8-GPC, or m8-N+GPC to explore the potential impact of pre-existing immunity to the viral vector vaccine. The findings indicated a decrease in protection, with 30% survival (m8-N), 60% survival (m8-N+GPC), and 70% survival (m8-GPC) against the lethal SFTSV challenge of 1 × 10^5^ TCID50. These results suggest that the rVAC-based vaccine provides protective immunity but may have limitations in immunizing individuals born prior to the 1980s, who may already have immunity to VACV (Yoshikawa et al., 2021).

### 4.6 Protein subunit vaccine

A protein subunit vaccine is a type of vaccine that contains harmless pieces of the pathogen (usually specific proteins) rather than the whole pathogen. These subunits are sufficient to stimulate an immune response without causing disease (Cox et al., 2015; Vartak and Sucheck, 2016; Costa et al., 2017; Kozak and Hu, 2023). By focusing on well-characterized antigens, protein subunit vaccines offer a high degree of safety and specificity. They eliminate the risks associated with live-attenuated or whole-inactivated pathogens while still eliciting robust immune responses when formulated with appropriate adjuvants.

There have been limited studies assessing the effectiveness of whole-inactivated SFTSV or SFTSV protein subunits as potential vaccines. Given the successful induction of humoral immunity by certain live vaccines mentioned earlier, it is expected that some level of efficacy would be observed if whole-inactivated SFTSV, SFTSV virus-like particles (VLPs), or SFTSV glycoprotein (Gn/Gc) subunits were used as vaccine components. However, this study specifically evaluated the efficacy of the SFTSV nonstructural protein (NSs) as a vaccine component (Grow et al., 2016). Immunocompetent C57BL/6 mice were immunized with 100 μg of purified recombinant NSs, produced by E. coli, using Freund’s complete adjuvant for the initial dose and Freund’s incomplete adjuvant for the booster. The mice were subsequently challenged with a pathogenic dose of SFTSV.

Despite adequate antigen delivery and adjuvant support, the viremia levels in the NSs-vaccinated mice did not show a significant reduction compared to those in control mice, indicating that NSs alone was ineffective in providing protective immunity or clearing the virus. The rationale for selecting NSs likely stemmed from its known role in antagonizing host innate immune responses, such as suppressing IFN-β production and modulating inflammatory signaling pathways. Nevertheless, its poor performance in eliciting protective immunity highlights the limitations of targeting nonstructural proteins that may not be naturally accessible to the immune system during infection.

Given the limited success of NSs, alternative structural proteins, such as the envelope glycoproteins Gn and Gc, warrant further investigation as subunit vaccine candidates. These glycoproteins are directly involved in host cell attachment and viral entry and have been shown to induce potent neutralizing antibody responses in other bunyavirus and phlebovirus systems. Studies using SFTSV VLPs or recombinant Gn/Gc subunits have demonstrated promising immunogenicity profiles in animal models, eliciting both humoral and cellular immune responses. For instance, immunization with SFTSV VLPs has been reported to stimulate neutralizing antibodies and confer protection in challenge models, suggesting their potential as effective subunit vaccines.

In addition, combining multiple viral antigens (e.g., Gn, Gc, and N) in a multivalent formulation may further enhance protective efficacy, especially when paired with advanced adjuvants such as toll-like receptor agonists, saponin-based compounds (e.g., Matrix-M), or nanoparticle carriers. These formulations could be designed to promote a balanced Th1/Th2 response and long-term immune memory.

### 4.7 mRNA vaccines

mRNA vaccines are a type of vaccine that use messenger RNA (mRNA) to instruct cells in the body to produce a protein that triggers an immune response. This protein is typically a harmless piece of the target pathogen, such as a surface glycoprotein, which the immune system can recognize and remember. Once the protein is synthesized inside the host cells, it is presented on the cell surface, prompting the immune system to generate both antibody and T-cell responses. This process mimics a natural infection and prepares the immune system to respond rapidly and effectively upon encountering the actual pathogen in the future.

This technology is relatively new in the field of vaccinology and has been notably used in the development of COVID-19 vaccines, such as those by Pfizer-BioNTech and Moderna. These mRNA vaccines demonstrated high efficacy and favorable safety profiles, leading to their rapid deployment and widespread use during the pandemic. The success of these vaccines validated mRNA platforms as a transformative tool in infectious disease prevention, stimulating a surge of interest in applying mRNA vaccine technology to other emerging and re-emerging infectious diseases (Pardi et al., 2018a; Baden et al., 2021; Roozen et al., 2024).

This study developed a lipid nanoparticle (LNP)-encapsulated mRNA vaccine expressing the SFTSV Gn protein (mRNA-Gn), which successfully elicited strong neutralizing antibody production and Gn-specific T-cell responses. Vaccinated mice exhibited complete protection against lethal SFTSV challenge, underscoring the vaccine’s promising potential as a protective measure against SFTSV infection. A key advantage of mRNA vaccines lies in their rapid and flexible manufacturing processes, enabling swift adaptation to emerging viral strains an essential feature for responding to future outbreaks of infectious diseases like SFTSV.

Beyond its robust immunogenicity and protective efficacy, the mRNA-Gn vaccine demonstrated excellent safety and tolerability in preclinical models, with no evidence of vaccine-associated enhancement or toxicity. Notably, it induced a balanced immune response, involving both potent humoral immunity and cellular responses. CD4^+^ and CD8^+^ T cell activation indicated the vaccine’s ability to promote both helper and cytotoxic immune mechanisms crucial for viral clearance and long-term protection.

The LNP formulation was instrumental in achieving this efficacy by protecting the mRNA from degradation, facilitating cellular uptake, and enhancing antigen expression *in vivo*. This highlights the critical role of delivery systems in maximizing mRNA vaccine performance. Similar strategies have been applied in small-molecule drug development, such as the nanoformulation of niclosamide with cholic acids, which markedly enhanced its systemic delivery and therapeutic efficacy against SARS-CoV-2 (Zhai et al., 2023). Furthermore, the modular nature of the LNP-mRNA platform allows for multivalent vaccine designs that could incorporate additional SFTSV antigens or target related phleboviruses, potentially broadening protective coverage.

Given the high fatality rate and the absence of licensed vaccines or therapeutics for SFTSV, this mRNA-Gn vaccine represents a significant advancement toward outbreak preparedness and public health response. Future studies will need to assess the durability of the induced immunity, performance in larger animal models, and readiness for clinical application. If validated, this vaccine could serve as both a preventive solution in endemic areas and a rapid-deployment tool during emerging outbreaks (Kim et al., 2023).

The present study introduces messenger RNA (mRNA) vaccine candidates, namely sGn-H and sGn-H-FT, both of which exhibit remarkable immunogenicity and the ability to provide protection *in vivo*. Mice that were immunized with either the sGn-H or sGn-H-FT mRNA lipid nanoparticle (LNP) vaccine showed robust production of total antibodies and neutralizing antibodies (NAbs) against sGn-H. Notably, NAb levels remained consistently high over an extended period, indicating the durability of the immune response. Furthermore, mice immunized with the sGn-H or sGn-H-FT mRNA LNP vaccine were completely protected from a lethal dose of SFTSV, with no fatalities observed. These findings underscore the potential of sGn-H and sGn-H-FT as vaccine antigen candidates capable of conferring protective immunity against SFTSV infection.

In addition to their strong humoral responses, these mRNA vaccines also stimulated significant cellular immune responses, including activation of both CD4^+^ helper T cells and CD8^+^ cytotoxic T lymphocytes, which play crucial roles in clearing viral infections and contributing to long-term protection. Immunophenotyping of splenocytes from vaccinated mice revealed increased cytokine production, such as IFN-γ and TNF-α, indicative of a Th1-biased immune response that is typically associated with effective antiviral activity.

The use of the LNP delivery system was instrumental in ensuring efficient intracellular delivery and expression of the mRNA constructs. The sGn-H-FT variant, which includes a furin cleavage site and a trimerization domain, was specifically designed to mimic the native conformation of the viral envelope protein, potentially enhancing the breadth and affinity of the antibody response. Structural optimization of antigens like sGn-H-FT may improve immunogenicity by promoting better epitope presentation and stability.

Taken together, the results support the advancement of both sGn-H and sGn-H-FT mRNA vaccines toward further preclinical development. Their ability to induce potent, durable immune responses and confer complete protection in a lethal challenge model highlights their promise as next-generation vaccine candidates for preventing SFTSV infection. Future research should aim to evaluate their efficacy in larger animal models, assess long-term immune memory, and explore scalable manufacturing processes in preparation for potential clinical trials (Kim et al., 2023).

## 5 Antiviral treatment against SFTSV

### 5.1 RNA-dependent RNA polymerase inhibitors

#### 5.1.1 Ribavirin

Previous study revealed that when ribavirin was applied both prior to and during SFTSV infection, the IC_99_ values measured were 263 μM for Vero cells, 83 μM for Huh7 cells, and 78 μM for U2OS cells, highlighting its potential antiviral activity (Shimojima et al., 2014). The antiviral effect of ribavirin was significantly reduced when Vero cells were treated three days after SFTSV inoculation, indicating that ribavirin may be most effective as a post-exposure prophylactic agent and may also serve as a component of combination therapy for SFTSV treatment (Shimojima et al., 2014). Ribavirin exhibits antiviral properties through both direct and indirect mechanisms. Direct actions encompass lethal mutagenesis, suppression of polymerase activity, and interference with RNA capping, while indirect effects involve immune system modulation and the inhibition of inosine monophosphate dehydrogenase (Graci and Cameron, 2006). Ribavirin’s antiviral activity against SFTSV was further confirmed in a study using a Korean strain, where treatment of infected Vero cells at 24- and 48-hours post-inoculation resulted in an IC50 range of 3.69–8.72 μg/mL (Lee et al., 2016). Ribavirin was found to reduce SFTSV replication even with differing viral strains and treatment conditions, implying its broad antiviral activity for at least 48 hours following SFTSV inoculation. Ribavirin showed no significant reduction in viral production when added to cells three days post-SFTSV infection, suggesting limited efficacy in fully infected cells or symptomatic patients. This aligns with clinical data showing high viral loads upon hospital admission and no improvement in patient outcomes with ribavirin treatment (Shimojima et al., 2015). In a 2019 observational study, ribavirin treatment showed no meaningful effect on mortality or recovery of selected laboratory indices compared to untreated patients (Zhang et al., 2021). Ribavirin showed effective antiviral activity against SFTSV *in vitro*, with Shimojima et al. recommending its use as a preventive treatment. Despite lower sensitivity in Vero cells, *in vivo* concentrations achieved in humans suggest ribavirin could still have substantial therapeutic effects, warranting further evaluation in suitable animal models (Shimojima et al., 2014). Bone marrow suppression can induce dose-related hemolytic anemia, which is typically reversible, and may also raise levels of uric acid, iron, and bilirubin in the plasma (Kowdley, 2005). These compounds are contraindicated in pregnancy because of their potential to cause birth defects and genetic mutations (Sakran et al., 2021).

#### 5.1.2 Favipiravir

Favipiravir (T-705) is an effective broad-spectrum antiviral drug first approved in Japan for treating novel and reemerging influenza viruses (Shiraki and Daikoku, 2020). It is effective against a broad range of viral pathogens, including influenza viruses, West Nile virus, yellow fever virus, flaviviruses, arenaviruses, bunyaviruses, alphaviruses, enteroviruses, and Rift Valley fever virus, and is recognized as a potential treatment for influenza and other RNA virus infections (Furuta et al., 2009). T-705 is converted by host enzymes into its active form, T-705 ribofuranosyltriphosphate. The active form inhibits viral RNA-dependent RNA polymerase. It may incorporate into viral RNA, causing lethal mutagenesis or chain termination. T-705 selectively targets viral polymerase without affecting host nucleic acid synthesis (Furuta et al., 2009). Its potential as a therapeutic candidate for influenza and other RNA viral infections has led to evaluations of its effectiveness against SFTSV in both *in vitro* studies (Tani et al., 2016; Baba et al., 2017) and *in vivo* experiments (Tani et al., 2016; Gowen et al., 2017; Smee et al., 2018). The IC_90_ of favipiravir (22 μM) in Vero cells (Tani et al., 2016) was considerably lower than that of ribavirin (263 μM) (Shimojima et al., 2014). Favipiravir treatment resulted in undetectable serum viral loads in SFTSV-infected mice after two days (Tani et al., 2016). In a comparative study, favipiravir treatment resulted in complete survival of bunyavirus-infected mice, in contrast to the ribavirin group, which suffered a high mortality rate (Gowen et al., 2017), indicating the promising protective efficacy of favipiravir against SFTSV.

Favipiravir has been tested in clinical trials worldwide, including RCTs, small, randomized studies, case series, and observational trials. While the research reports vary, the majority support the drug’s efficacy.

A study of two SFTSV patients treated with favipiravir (1600 mg twice on day 1, followed by 600 mg twice daily for 4 days) between days 4 and 11 post-onset resulted in complete recovery (Song et al., 2020). In a randomized clinical trial of 145 patients, favipiravir (1800 mg twice on day 1, then 1000 mg twice daily for 5 days) showed better viral clearance and disease progression outcomes compared to the control group (standard supportive care) (Li et al., 2021). In an uncontrolled, non-randomized, multicenter clinical study involving 23 SFTSV patients, favipiravir (1800 mg orally twice on day 1, followed by 800 mg twice daily for 7–14 days) was associated with a higher 2-day recovery rate compared to previous studies from Japan (Suemori et al., 2021), although the absence of a control group and potential biases limit definitive conclusions.

Favipiravir is safe for human use, as it does not interfere with mammalian DNA or RNA synthesis and is non-toxic to cells. Furthermore, it demonstrates superior effectiveness and safety profiles compared to ribavirin.

#### 5.1.3 Fludarabine

Fludarabine, a synthetic analogue of the adenine nucleoside, is predominantly used in the first line and second-line treatment regimens for a variety of lymphoproliferative malignancies (Leporrier, 2004). It was first synthesized by Hewson and Montgomery in 1969 (Montgomery and Hewson, 1969). The results showed that fludarabine inhibited SFTSV replication in a dose-dependent manner in HMC3 microglial cells (IC50 = 0.42 ± 0.01 μM) and U251 MG astrocytes (IC50 = 0.28 ± 0.17 μM). The researchers assessed viral replication, proliferation, and protein expression, concluding that fludarabine had potent antiviral effects in both U251 and HMC3 nerve cells. In line with these results, a similar study also reported that fludarabine effectively combats other positive-strand RNA viruses, such as Zika virus (ZIKV) and Enterovirus A71 (Gao et al., 2021).

Despite its antiviral efficacy, fludarabine is associated with certain cytotoxic effects and clinical side effects. The most frequent side effects include myelosuppression, lymphocytopenia, and secondary infections, along with gastrointestinal symptoms like increased liver enzymes, vomiting, and nausea. SFTSV patients, who commonly suffer from liver and kidney damage and organ failure, should be closely monitored (Ding et al., 2008).

#### 5.1.4 Calcium channel inhibitors

Calcium ions (Ca²^+^) serve as essential intracellular messengers with broad and complex roles in modulating numerous cellular pathways (Bagur and Hajnóczky, 2017). Their involvement in viral infections is believed to stem from their regulation of calcium-dependent cytokines necessary for viral entry and replication (Zhou et al., 2009). The antiviral effects of calcium channel blockers (CCBs) have been increasingly reported against several highly pathogenic viruses, including Japanese encephalitis virus, West Nile virus (WNV), Junin virus, Marburg virus, and Ebola virus. These CCBs have been shown to inhibit viral entry in the case of Ebola and interfere with fusion and entry in Junin virus infections (Lavanya et al., 2013; Gehring et al., 2014; Sakurai et al., 2015). Although the stages of viral inhibition by CCBs have been identified, the precise molecular mechanisms remain unclear. This may be due to the complex regulatory functions of intracellular Ca²^+^, which influences multiple cellular processes such as membrane fusion, endocytosis, and gene transcription through the modulation of Ca²^+^-dependent proteins.

A screening of 700 FDA-approved drugs revealed that the calcium channel blockers (CCBs) benidipine hydrochloride and nifedipine significantly inhibited SFTSV replication *in vitro* by disrupting viral internalization and impairing genome replication during the post-entry phase (Li et al., 2019). Notably, these compounds did not interfere with viral binding, fusion, or budding. The *in vitro* findings suggested that both CCBs reduce virus-induced Ca²^+^ influx, thereby inhibiting viral replication. Further investigation in C57BL/6 and humanized mouse models demonstrated that treatment with benidipine hydrochloride or nifedipine led to decreased viral load, elevated platelet counts, and reduced mortality in the humanized model.

Nifedipine, a commonly prescribed medication for hypertension and atherosclerosis in China, has recently gained attention for its potential therapeutic effects in severe fever with thrombocytopenia syndrome. In a retrospective clinical study involving 2,087 patients, Li et al. categorized individuals into three groups: 83 patients who received nifedipine both prior to admission and throughout hospitalization, 48 patients who were administered nifedipine only before admission, and 249 patients who had no exposure to the drug (Li et al., 2019). Notably, the fatality rate in the group treated with nifedipine during hospitalization was significantly reduced to 3.6%, representing over a five-fold decrease compared to both the non-treated group (19.7%) and those who discontinued nifedipine after admission (20.8%). Among patients with high viral loads (>10^6^ copies/mL), the case fatality rate dropped to 2.4% in the nifedipine-treated group, compared to 29.0% and 34.5% in the general and previously treated-only groups, respectively. Additionally, the incidence of hematemesis a hemorrhagic symptom closely linked to mortality was markedly lower in nifedipine-treated patients. Complementing the clinical findings, the study also demonstrated that both nifedipine and benidipine hydrochloride exhibited antiviral activity *in vitro* and in animal models. Importantly, nifedipine administration was associated with enhanced viral clearance and improved clinical outcomes.

#### 5.1.5 Caffeic acid

Caffeic acid (CA) and its derivatives are widely present in nature and frequently used in creating biologically active substances (Muhammad Abdul Kadar et al., 2021). Their structures are highly modifiable, enabling the development of esters, amides, and polymer-based derivatives with varied and potent biological functions. Studies conducted both in laboratories and animal models have demonstrated that caffeic acid (CA) provides a broad range of health benefits, including reducing inflammation, inhibiting cell proliferation, easing anxiety, protecting nerve cells, fighting microbes, modulating the immune system, and combating oxidative stress (Ogawa et al., 2018; Ogawa et al., 2021; Zielińska et al., 2021). Previous studies have reported that the selectivity index (SI) of caffeic acid (CA) against hepatitis B virus (HBV) is 128, while a related compound, a phenethyl ester derivative of CA, exhibits an SI of 17.9 against hepatitis C virus (HCV) (Wang et al., 2009; Shen et al., 2013). Although CA exhibits antiviral activity against several viruses, there is no definitive proof that this effect is due to its direct interaction with their viral particles. For HCV and IAV, CA appears to inhibit the early stages of infection, including viral entry, suggesting a mechanism like its action on SFTSV. Conversely, CA suppresses HBV infection mainly at the genome replication phase. The precise target of CA in HSV-1 infection remains unknown. Thus, additional research is needed to determine whether CA directly affects the viral particles of these pathogens. In their study, Ogawa et al. found that pre-incubating SFTSV with caffeic acid (CA) markedly reduced the virus’s ability to attach to host cells, while CA treatment of already infected cells showed only moderate antiviral activity. These results suggest that CA may block viral transmission by targeting the virus directly rather than the host cells. Their findings highlight CA as a promising candidate for both the prevention and treatment of SFTSV and offer a meaningful foundation for future drug development efforts targeting the virus (Ogawa et al., 2018).

### 5.2 Quinoline analogues

#### 5.2.1 Amodiaquine

Amodiaquine is an antimalarial medication belonging to the 4-aminoquinoline class, closely related to chloroquine. It has been extensively used in clinical settings for both treating and preventing malaria and is an officially approved therapeutic option. Beyond its primary uses, it is also prescribed for conditions such as liver amebiasis, infections from *Schistosoma haematobium* and *Schistosoma mansoni*, and connective tissue diseases. Furthermore, it has proven beneficial in treating photosensitivity disorders, including solar erythema. Additionally, it has demonstrated antiviral activity against the Ebola virus (DeWald et al., 2019), dengue virus and Zika virus (Han et al., 2018). However, the precise mechanisms through which it exerts inhibitory effects on malaria and these viral infections have yet to be fully elucidated. In their study, Baba et al. evaluated amodiaquine and halogen-substituted analogues (fluorine, bromine, iodine) for anti-SFTSV activity *in vitro (* Baba et al., 2017). o screen for anti-SFTSV drugs, SFTSV-infected cells were treated with varying concentrations of the compounds and cultured under these conditions. Intracellular SFTSV RNA levels were then quantified using specific TaqMan primers/probes and a real-time RT-PCR assay. All showed inhibitory effects, with IC50 values of 36.6 μM (fluorine), 31.1 μM (bromine), and 15.6 μM (iodine). Amodiaquine was identified as a selective SFTSV replication inhibitor, with a CC50 >100 μM and IC50 of 19.1 μM lower than ribavirin (40.1 μM) and favipiravir (25.0 μM).

#### 5.2.2 Chloroquine/hydroxychloroquine

Chloroquine and hydroxychloroquine, classified as quinoline derivatives, were originally developed for antimalarial therapy. Nevertheless, advances in understanding their pharmacological mechanisms have led to their broader use in managing infectious diseases, autoimmune disorders, and various malignancies (Rainsford et al., 2015). Chloroquine and hydroxychloroquine have been shown through in-depth investigations to exert antiviral effects against several viruses, including coronaviruses (Persoons et al., 2021), influenza virus and Crimean-Congo hemorrhagic fever (CCHF) virus (Ferraris et al., 2015). Additionally, both drugs have demonstrated substantial clinical efficacy in the management of rheumatologic conditions such as systemic lupus erythematosus and rheumatoid arthritis.

#### 5.2.3 Hexachlorophane

Hexachlorophene, an organochlorine compound, was once widely used as a topical antibacterial and antifungal agent, functioning as a disinfectant, surgical scrub, and also as a bactericide, fungicide, and pesticide in agriculture (Kimbrough, 1973). Beyond these applications, it also exhibited notable antiviral activity against a range of RNA and DNA viruses. Studies have shown that disinfectants containing hexachlorophene were effective against rhinovirus (when combined with ethyl alcohol) and rotavirus, particularly at concentrations of 0.75% hexachlorophene or 0.1% hexachlorophene with 70% isopropyl alcohol (Hendley et al., 1978; Sattar et al., 1983). Furthermore, hexachlorophene was found to inhibit the 3C-like protease activity of the severe acute respiratory syndrome coronavirus, as well as suppress the *in vitro* replication of other coronaviruses, including murine hepatitis virus, bovine coronavirus, and human enteric coronavirus (Hsu et al., 2004; Cao et al., 2015). Additionally, it demonstrated antiviral properties against polyomaviruses such as BK virus and Simian Virus 40 by inhibiting the ATPase activity of their large tumor antigen, thereby disrupting viral replication (Seguin et al., 2012). Hexachlorophene demonstrated potent antiviral activity against SFTSV, significantly reducing viral RNA levels by over 2-log10 and achieving complete (100%) plaque reduction at a 10 µM concentration, which remained below it’s CC50 (Yuan et al., 2019). With an IC50 ranging from 1.3 to 2.6 µM, hexachlorophene exhibited greater potency against SFTSV compared to other reported antiviral compounds, including caffeic acid (180 µM), ribavirin (40.1 µM), favipiravir (25.0 µM), amodiaquine (19.1 µM), and 2′-fluoro-2′-deoxycytidine (3.7 µM) (Baba et al., 2017; Ogawa et al., 2018; Smee et al., 2018).

### 5.3 Immunotherapy

#### 5.3.1 Glucocorticoids

Glucocorticoids are widely employed for their broad-spectrum therapeutic effects in managing acute infections, severe inflammation, asthma, and autoimmune disorders, as well as for mitigating post-inflammatory complications. In the context of severe SFTSV, a cytokine storm may be triggered, eliciting a hyperinflammatory response that can ultimately result in fatal outcomes (Yoo et al., 2021). Given this pathophysiological mechanism, glucocorticoids have been proposed as potential agents to suppress cytokine-mediated inflammation and alleviate clinical manifestations. Several case reports from international studies have documented the successful use of corticosteroids in combination with other therapeutic agents for treating SFTSV infection, including cases complicated by encephalopath**y** (Kim et al., 2013; Nakamura et al., 2018). Notably, pulse corticosteroid therapy has been administered in patients with SFTSV-associated encephalopathy, with findings suggesting that short-term glucocorticoid intervention may be beneficial during the early phase of neurological involvement. However, despite their capacity to promptly reduce inflammatory symptoms, glucocorticoids may increase susceptibility to secondary infections due to their immunosuppressive effects. Moreover, the current body of literature lacks robust evidence to support the routine use of glucocorticoids in SFTSV treatment. Further in-depth clinical and mechanistic studies are warranted to clarify their efficacy and safety in this context.

#### 5.3.2 Plasma exchange

Plasma exchange is a therapeutic blood purification technique that removes metabolic toxins and pathogenic factors via extracorporeal circulation. This intervention has been shown to restore internal homeostasis and improve coagulation function. It has been applied in the management of critically ill patients with severe sepsis, H7N9 influenza, and Ebola virus infections (Liu et al., 2015)**. I**n the context of SFTSV, a previous report noted that plasma exchange alone did not lead to clinical improvement in a critically ill patient. However, when combined with ribavirin, clinical improvement was observed in two patients with rapidly progressing SFTSV (Oh et al., 2014). These findings suggest that, for patients in the multiple organ dysfunction syndrome (MODS) stage of SFTSV, the combination of plasma exchange and ribavirin may serve as promising salvage therapy.

A recent study conducted in South Korea demonstrated that therapeutic plasma exchange (TPE) led to a marked reduction in serum viral load and rapid improvement in both clinical symptoms and laboratory parameters in patients with SFTSV, suggesting that TPE may help mitigate disease progression in rapidly deteriorating cases (Yoo et al., 2017). However, conflicting evidence exists, as a separate case report documented no reduction in viral load following TPE (Utsunomiya et al., 2022). These findings are limited by small sample sizes and the absence of rigorous control groups, highlighting the need for cautious interpretation. Consequently, the use of TPE should be guided by careful clinical evaluation, considering the specific manifestations and progression of each patient.

In addition to limited clinical evidence, the potential adverse effects of TPE must also be considered. The extracorporeal circulation of blood during the procedure may impose stress on cardiopulmonary function, particularly with repeated sessions. Moreover, anticoagulant use during plasma replacement can result in the depletion of coagulation factors, thereby compromising hemostatic function. TPE is typically administered during the early to middle phases of cytokine storm in severely or critically ill patients, many of whom may already exhibit multi-organ dysfunction. Therefore, patient selection for TPE should be based on a comprehensive clinical assessment, incorporating laboratory findings, imaging studies, and multidisciplinary clinical judgment to ensure appropriate and effective application.

### 5.4 Targeted therapy strategy

#### 5.4.1 Antibody therapy

Immunotherapeutic approaches targeting host inflammatory responses have also been explored. Tocilizumab, an anti-IL-6 receptor monoclonal antibody, has shown potential benefits in SFTSV patients with elevated IL-6 levels, both in case reports and clinical trials (Yoo et al., 2022; Ge et al., 2024).

MAb 4–5 is a recently identified human neutralizing monoclonal antibody that targets a surface-exposed, cross-reactive epitope within the N-terminal region of the SFTSV Gn glycoprotein, specifically binding to domain III (Wu et al., 2017). Although MAb 4–5 has demonstrated potent neutralizing activity *in vitro*, its *in vivo* efficacy remains to be established. Another promising candidate, Ab10, has shown therapeutic efficacy in a murine model of SFTSV infection (Kim et al., 2019). However, its neutralizing activity was evaluated only in Vero cells and limited to the Gangwon/Korea/2012 strain, underscoring the need for broader validation.

To develop novel antibody-based therapeutics, the extracellular domain of the SFTSV Gn glycoprotein (sGn) expressed in mammalian cells was used to immunize a camel. Peripheral blood mononuclear cells (PBMCs) from the immunized camel were subsequently employed to construct a variable heavy chain domain (VHH) antibody phage library. Following several rounds of bio panning against sGn, 23 nanobodies with potent *in vitro* neutralizing activity were identified. Among them, SNB02 an engineered VHH fused to a human Fc1 domain (designated SNB) exhibited strong neutralization capacity against SFTSV *in vitro* (Wu et al., 2020).

Collectively, these findings support the growing potential of antibody-based therapeutics as a critical component of future antiviral strategies. Continued development and *in vivo* validation of these agents are essential for expanding the arsenal against SFTSV and related emerging pathogens.

#### 5.4.2 Arginine

In 2018, a study utilizing metabolomics revealed that SFTSV infection induces disruptions in arginine metabolism. This alteration was found to correlate with thrombocytopenia and T lymphocyte dysfunction in affected patients (Li et al., 2018). Clinical trials subsequently demonstrated that arginine supplementation facilitated faster recovery, providing evidence for its potential therapeutic role. By examining the underlying metabolic and immunological processes, the study offered valuable insights into the pathophysiology of the disease, which could inform novel therapeutic approaches.

A comprehensive metabolomic analysis of 242 clinical cases showed marked changes in the metabolic profiles of SFTSV-infected individuals. Pathway enrichment analysis of the differentially regulated metabolites identified the arginine metabolic pathway as the most significantly altered. Arginine levels were found to be significantly reduced in infected patients. Further investigation revealed that the downregulation of arginine led to a decrease in platelet nitric oxide (Plt-NO) production, which in turn contributed to increased platelet activation and apoptosis. These findings suggest that arginine deficiency may play a crucial role in the development of coagulation abnormalities observed in SFTSV infection.

#### 5.4.3 IFN-γ

IFN-γ is the sole member of the type II interferons and plays a crucial role in the immune response by stimulating macrophages and dendritic cells, thereby enhancing direct antimicrobial activities through the modulation of antigen processing and presentation pathways. Initially, it was believed that activated T cells and natural killer (NK) cells were the primary sources of IFN-γ. However, it has since been demonstrated that macrophages and dendritic cells can also produce IFN-γ under certain conditions *in vitro*. IFN-γ exerts its antiviral effects by activating the STAT1 signaling pathway, which in turn induces the expression of several antiviral interferon-stimulating proteins, making it a key player in the defense against viral infections.

In a study by Ning et al., enzyme-linked immunosorbent assays (ELISA) revealed that SFTSV infection leads to a marked increase in serum IFN-γ levels in patients with SFTSV. *In vitro*, IFN-γ exhibited potent anti-SFTSV activity in cultured cells (Ning et al., 2019). The therapeutic potential of IFN-γ was further evaluated in a suckling mouse model, where pre-infection treatment with IFN-γ significantly reduced mortality, with approximately 25% of the mice surviving the infection. In contrast, all untreated mice succumbed to the virus within 13 days of SFTSV exposure. However, when IFN-γ was administered post-infection, it failed to protect the mice, and 100% of the animals died from the viral infection.

## 6 Conclusion

Significant advances in SFTSV research have clarified disease mechanisms and supported the development of vaccine and therapeutic candidates. Immunocompromised mouse models and non-human primates have provided critical preclinical insights. mRNA and viral vector vaccines have demonstrated strong immunogenicity, while antivirals such as favipiravir and monoclonal antibodies show therapeutic potential. Despite this progress, no approved treatment or vaccine currently exists, underscoring the need for continued investigation. Integrating molecular tools such as transcriptomics and proteomics with next-generation platforms may further enhance the efficacy of future interventions. A critical gap remains in understanding the durability of immunity following infection or vaccination, highlighting the need for studies to identify correlates of protection and evaluate long-term immune responses across diverse populations.
